# Lexical density, lexical diversity, and lexical sophistication in simultaneously interpreted texts: a cognitive perspective

**DOI:** 10.3389/fpsyg.2023.1276705

**Published:** 2023-10-02

**Authors:** Zhibo Liu, Juhua Dou

**Affiliations:** School of International Cooperation, Guangdong Polytechnic of Science and Technology, Zhuhai, China

**Keywords:** lexical density, lexical diversity, lexical sophistication, cognitive load, simultaneous interpreting, interpreting directionality, cognitive demands

## Abstract

Simultaneous interpreting (SI) is a cognitively demanding task that imposes a heavy cognitive load on interpreters. Interpreting into one’s native (A language) or non-native language (B language), known as interpreting directionality, involves different cognitive demands. The cognitive requirements of simultaneous interpreting as well as interpreting directionality affect the interpreting process and product. This current study focused on the lexical features of a specially designed corpus of United Nations Security Council speeches. The corpus included non-interpreted speeches in US English (SubCorpusE), and texts interpreted from Chinese into English (A-into-B interpreted texts, SubCorpusC-E) and from Russian into English (B-into-A interpreted texts, SubCorpusR-E). Ten measures were used to analyze the lexical features of each subcorpus in terms of lexical density, lexical diversity, and lexical sophistication. The three subcorpora were regrouped into two pairs for the two research questions: SubCorpusR-E versus SubCorpusE and SubCorpusR-E versus SubCorpusC-E. The results showed that the interpreted texts in SubCorpusR-E exhibited simpler vocabulary features than the non-interpreted texts in SubCorpusE. In addition, compared with the A-into-B interpreted texts, the B-into-A interpreted texts demonstrated simplified lexical characteristics. The lexical features of the interpreted texts reflect that experienced simultaneous interpreters consciously adopt a simplified vocabulary approach to manage the cognitive load during simultaneous interpreting. This study provides new insights into the cognitive aspects of simultaneous interpreting, the impact of directionality, and the role of lexical strategies. These findings have practical implications for interpreter training, professional growth, and maintaining interpreting quality in diverse settings.

## 1. Introduction

Simultaneous interpreting (SI) is an extremely intricate task that involves bilingual processing and cognitive coordination. It requires multiple concurrently performed sub-tasks such as perception and analysis of the source information; memorization and storage of the accessed information; activation, retrieval, and selection of linguistic and extralinguistic knowledge for comprehension and production; reconstruction and reformulation in the target language; and evaluation and modulation of the interpreted output (Zhu, 2021; Liu et al., 2022). The properties of SI, such as complexity, time constraints, concurrent multitasking, simultaneity, make SI a cognitively demanding task compared with prepared non-interpreted speeches.

Another factor that makes SI a cognitively demanding task is directionality. This refers to the direction in which the language is translated or interpreted: either into one’s native language (first language or dominant language) or into one’s non-native language (second language or non-dominant language) (Beeby, 1998). AIIC (2006), the International Association of Conference Interpreters, adopted the terms “A language” and “B language” to elucidate the interpreting directionality issue. Each interpreting direction has different cognitive demands for interpreters and requires different cognitive efforts from them, resulting in different interpreting performance in terms of delivery, accuracy, output quality. The common assumption holds that working into one’s B language is more difficult than working into one’s A language, because the A-into-B direction imposes a heavier cognitive load on interpreters than the B-into-A direction (Liao and Chan, 2016; Chou et al., 2021).

Lexical features encompass a range of linguistic elements, such as lexical choices, word frequency, and lexical richness. By analyzing lexical patterns in the output of simultaneous interpreters, researchers can gain valuable insights into how interpreters adapt their language use to optimize their cognitive resources and manage their cognitive load of real-time interpretation. This examination not only sheds light on the strategies used by interpreters to effectively handle the cognitive load involved in interpreting tasks but also has pedagogical implications for interpreter training and performance enhancement. It enables the identification of effective language techniques that can improve interpreter performance and mitigate cognitive fatigue.

Conference interpreters working for the United Nations (UN) exhibit high levels of bilingual proficiency, exceptional interpreting skills, and extensive experience (Cheung, 2019). Exploring how these skilled interpreters handle their demanding cognitive task and whether their interpreting performance is influenced by directionality warrants thorough investigation. To address these questions, this study analyzed the lexical features of three subcorpora, namely SubCorpusE, SubCorpusC-E, SubCorpusR-E. Measures such as lexical density, lexical diversity, and lexical sophistication were employed as indicators to examine how these interpreters adeptly manage cognitive overload during SI.

## 2. Literature review

### 2.1. Working memory and simultaneous interpreting

Working memory is a central aspect of cognitive processes and plays a crucial role in successful SI performance. It is a cognitive system that can hold and manipulate information for short periods and facilitate the interaction between new information entering the mind and knowledge stored in long-term memory by means of short-term storage and processing resources (Pöchhacker, 2016). Early theoretical and empirical studies have shown that human beings have limited working memory capacity (Welford, 1952; Broadbent, 1958). This restriction lies not only in the number of simultaneous operations it can perform but also in the amount of information it can retain for processing (Seeber, 2011). Due to these limitations, the attentional control of working memory, i.e., focusing attention, dividing attention, and switching attention (Baddeley, 2002), is also constrained (Cowan, 1999). This makes it difficult for simultaneous interpreters to execute multiple operations, process information items that surpass the predetermined number of information clusters, and efficiently coordinate attentional resources (Liu et al., 2004).

In addition to the two limitations concerning the quantity of task operations and information to be processed, working memory capacity is constrained by temporal restrictions (Pöchhacker, 2016). According to Cowan (1995) model, activated memories will start to fade within a relatively short period, typically between 10 and 30 s, if they are not refreshed. To prevent the decay of these memory traces, they must be revived through mental repetition, which involves repeating the information silently and is used to facilitate memorization. This technique allows the information to be held in working memory for a longer period, providing more time to process and integrate it with other information. However, the parallelism and simultaneity of SI render such mental rehearsal impossible, making memory refreshment extremely difficult. As a result, the time constraints on working memory, the subsequent linguistic input that requires longer storage, and the extralinguistic information that requires deep processing are likely to severely tax and greatly exceed simultaneous interpreters’ working memory capacity, forcing them to expend a substantial amount of mental effort and resources to successfully accomplish the task.

### 2.2. Effort model

The effort model (Gile, 2008) is a conceptual framework that views SI as a complex cognitive process involving multiple cognitive operations. According to Gile (2008), these cognitive operations can be classified into three main efforts: listening and analysis effort to facilitate the comprehension of the source speech; production effort to help interpreters produce a target speech, including the effort spent on self-observation, self-evaluation, and self-modification; and memory effort, which is responsible for managing and controlling the storage and retrieval of information associated with the source and target speeches. A fourth category, known as coordination effort, functions to manage the allocation of attention and shift of focus between the three core efforts. In other words, it ensures that the interpreter is able to coordinate the various cognitive processes involved in SI in an efficient manner and maintain the real-time flow of interpretation.

On the basis of this effort model, Gile (2015) further proposed the “competition hypothesis,” which assumes that the three core efforts compete with each other. This means that adding or overinvesting in one type of effort will weaken the other processing components. For example, when cognitive resources are allocated to comprehension of the source speech, the rest of the cognitive components, i.e., production of the target speech, will to some degree be impaired, because the attentional resources for memory, production, and coordination are extracted for better comprehension and analysis of the source speech. Therefore, it is expected that the outcome of one effort will be at the expense of the others. This is generally reflected by simultaneous interpreters’ self-report that working in simultaneity and parallelism poses a challenge in terms of equal allocation of effort during SI.

Another theory suggesting that simultaneous interpreters work under a heavy cognitive burden is the “tightrope hypothesis” (Gile, 2008), which assumes that the total cognitive demands of the SI task tend to approach the maximum level of the interpreter’s available cognitive capacity. This means that any increase in processing demands or any occurrence of cognitive resource mismanagement may lead to overload or a decrease in attention in one of the efforts, which stretches and strains the interpreter and consequently leads to a change or even a decline in interpretation quality.

### 2.3. Which is more cognitively demanding: SI or non-interpreted speeches?

The effort model (Gile, 2008) suggests that SI requires great mental effort because the three core efforts are not automatically activated but instead require attentional resources and energy to be devoted to them. Memory effort in particular is unlikely to be automatic because it constantly operates by storing and retrieving information, differentiating storage for the source and target speeches, activating or inhibiting memory, and seeking associations with the mental lexical and syntactic representations of the source and target languages. All of these processes are achieved through attentional resources and are not typically involved in non-interpreted speeches (Gile, 2015). In other words, compared with non-interpreted speeches, SI involves higher cognitive demands as interpreters must actively and consciously engage in all three efforts simultaneously rather than relying on automatic processing.

Empirical studies have also confirmed that interpreting tasks impose a higher cognitive load. Hyönä et al. (1995) conducted a study comparing the processing load of SI with that of non-interpreted language tasks, such as listening to and repeating an auditory text by measuring changes in participants’ pupil size when they were performing the tasks. They found that pupillary response was a reliable indicator of cognitive load. Based on the changes in pupillary response to the different tasks, this study revealed significant differences in cognitive load between the SI task and the non-interpreted language tasks.

Plevoets and Defrancq (2018) discovered that compared with non-interpreted Dutch texts, interpreted Dutch texts exhibited a significantly higher occurrence of vocalized hesitation, “uh(m),” indicating disfluency and difficulties during interpretation. This suggests that interpreting tends to impose higher cognitive demands than non-interpreted speech (Goldman-Eisler, 1967; Setton, 1999). Additionally, their study revealed that one cognitive trigger, the lexical density of the source texts, was positively correlated with the occurrence of “uh(m)” in the interpreted texts. However, in the non-interpreted texts, cognitive triggers (lexical density of the source texts and occurrence of numbers) did not increase the frequency of “uh(m)”; instead, the number of “uh(m)” decreased with the occurrence of numbers. These triggers, which are commonly believed to cause cognitive load, not only do not increase disfluency but actually decrease it in the non-interpreted texts. This can be explained by the fact that non-interpreted speeches are often scripted, rehearsed, and well prepared (Plevoets and Defrancq, 2018), which greatly reduces the cognitive load of speakers. Interpreters, in contrast, tend to speak spontaneously without preparation, which means that they bear a higher cognitive load when exposed to a large influx of new information.

The theoretical and empirical studies reviewed above indicate that high cognitive demands are the inherent property of SI. Therefore, it is reasonable to assume that simultaneous interpreters may resort to certain “shortcuts” (Seeber, 2011) or use specific processing tactics to manage the intrinsic constraints of this challenging cognitive task (Riccardi, 1998), conserve processing capacity, and minimize the overall cognitive load.

### 2.4. Which direction results in a heavier cognitive load: A into B or B into A?

Interpreting from one’s A language into one’s B language and from one’s B language into one’s A language require different levels of cognitive effort. It is widely recognized that A-into-B interpreting requires more cognitive effort to construct equivalent linguistic and cultural expressions in one’s B language (e.g., Donovan, 2003, 2005) even if the A language input load is negligible. However, while it may be easier to produce linguistically and culturally equivalent expressions in one’s A language (Le Féal, 2003), interpreters working from their B language to their A language need to invest more effort to overcome the B language input load and ensure accurate comprehension and dependable interpretation (Denissenko, 1989). This means that interpreters experience more output load in the A-into-B direction and more input load in the B-into-A direction of interpretation. Interpreters’ performance in each direction also varies based on their own bilingual proficiency, which is referred to as the “asymmetry effect” or “directionality effect” (Chou et al., 2021). Empirical studies (Darò et al., 1996; Mead, 2000; Chen, 2020; Chou et al., 2021; Lu et al., 2023) have confirmed the existence of the directionality effect but have not reached a consensus on which interpreting direction imposes a higher cognitive load on interpreters or yields better or worse performance.

A common assumption is that shifting from the dominant language to the non-dominant language is more cognitively demanding than shifting in the reverse direction (Liao and Chan, 2016; Chou et al., 2021). This is supported by a number of empirical studies. The experiments conducted by Hyönä et al. (1995) revealed that when their participants produced lexical words in a non-native language, they showed increased pupil dilation compared with producing words in their native language. As pupil size is known to increase with cognitive load, it can be inferred that interpreters face higher processing demands when interpreting into their B language. Rinne et al. (2000) measured the brain activation patterns of professional interpreters and found that there was more widespread activation during interpretation into their B language. As the extent to which the brain activates is positively associated with the cognitive load placed on the brain, this indicates that interpreting in this direction is a more cognitively challenging task.

This experimental conclusion is manifested in interpreters’ performance. Generally, when interpreters perform worse in one direction than in the other, it suggests that their cognitive load in that direction is higher. Researchers have observed that interpreters respond to lexical words more slowly when working from their A language into their B language than when working from their B language into their A language (De Bot, 2000). In terms of syntactic processing, generating B language syntax has been thought to be less automatic and often necessitates deliberate monitoring (Bialystok, 1994). Studies have also shown that less experienced interpreters tend to be disfluent (Chou et al., 2021), presenting more filled pauses (Mead, 2000) and ungrammatical pauses (Fu, 2013) when interpreting into their B language. Darò et al. (1996) discovered that interpreters made more important errors that resulted in information loss when interpreting challenging texts from their A language into their B language. The lexical and syntactic challenges related to B language processing, the disfluency features and lack of completeness arising from interpreting into one’s B language, interpreters’ self-reported feelings of decreased confidence and even reluctance to work into their non-native language (Donovan, 2004), their consciously searching for and monitoring corresponding expressions in their B language, as well as audience’s perception of unsatisfactory interpreting quality due to the presence of a non-native accent (Cheung, 2015), indicate that interpreting into one’s B language requires a higher level of cognitive resources than interpreting into one’s A language.

Most studies seem to acknowledge that A-into-B interpreting involves higher cognitive demands and greater processing difficulty than B-into-A interpreting. However, there are still advocates for interpreting in this direction. Studies have shown that interpreting from one’s A language achieves a higher level of accuracy and completeness (Rinne et al., 2000; Lu et al., 2023) because of the minimal mental effort required for understanding the source speech in one’s native language. This, in turn, facilitates better quality of the interpreting output (Denissenko, 1989) and smooth delivery (Chen, 2020). In addition, many studies have indicated that B-into-A interpreting lacks accuracy and information completeness (Chen, 2020; Chou et al., 2021; Bu and Li, 2022) and produces more repairs (Fu, 2013; Song and Cheung, 2019) due to the higher input load involved in comprehending one’s B language.

The impact of directionality on expert interpreters appears to be complex, with mixed findings. Some studies have revealed better performance in the B-into-A interpreting direction (Al-Salman and Al-Khanji, 2002; Mead, 2005), particularly with respect to information completeness (Chang and Schallert, 2007). Other studies have reported higher information completeness in the A-into-B direction (Rinne et al., 2000). Finally, some research has shown no effect of directionality on information completeness but a slightly better delivery rate when interpreting into one’s B language (Nicodemus and Emmorey, 2015).

### 2.5. Lexical density, lexical diversity, and lexical sophistication

Lexical features are normally operationalized in terms of three indicators: lexical density, lexical diversity, and lexical sophistication. Lexical density is the ratio of the number of content words (lexical items) to the sum of content words and function words (grammatical items) (Ure, 1971). It is used to measure the lexical richness of oral and written discourse from the perspective of information-carrying capacity. The greater the lexical density, the more information the discourse carries, which leads to a higher cognitive load for information processing. Plevoets and Defrancq (2018) found lexical density in interpreted texts to be a strong factor of the occurrence of the disfluency marker “uh(m),” which suggests that interpreters experience cognitive load during interpretation.

Lexical diversity refers to the variation in vocabulary use within a text. It is measured by the value of the type-token ratio (TTR). A high score indicates an extensive and varied use of vocabulary with less repetition, while a low score suggests a high level of lexical repetitiveness and a limited range of vocabulary choices. However, the TTR is not always a reliable index to measure and compare lexical diversity across texts. One limitation is that it depends on the length of the text. For example, a short text with a limited number of unique words may have a high TTR because the text contains few tokens, resulting in a high proportion of distinct words. However, a long text with more words may have a low TTR, not only because of the number of repeated words but also because the occurrence of new words does not necessarily increase with the increase in the total number of words. Therefore, when interpreting TTR values, it is important to take into consideration the length of the analyzed text. One typical approach is to normalize it, for example by calculating the TTR value for every *n* tokens.

Lexical sophistication refers to the use of advanced vocabulary. It is typically assessed by using various metrics such as mean word length or the frequency of rare, infrequent, or academic words. Lexical sophistication can reflect both the degree of the cognitive demands required by a task and an interpreter’s capability of effectively managing his or her cognitive load while dealing with multiple tasks. Typically, higher lexical sophistication suggests lower cognitive demands and stronger cognitive abilities in handling the load, and vice versa.

### 2.6. Lexical features in interpreted texts

Research has indicated that interpreted texts display distinct lexical features compared with source texts, which is largely attributed to interpreters’ management and mitigation of their cognitive load during the interpreting process. According to Lv and Liang (2019), consecutive interpreting is associated with a higher cognitive load than SI, resulting in more salient vocabulary simplification features in consecutively interpreted texts in terms of lexical density, lexical repetitiveness, and lexical complexity than in simultaneously interpreted texts. While Lv and Liang (2019) challenged the traditional notion that SI is the most cognitively demanding task, their findings strongly support the idea that a high cognitive load results in lexical simplification. They also demonstrated that simultaneously interpreted texts, despite delivering more informative content and exhibiting higher lexical complexity than the original speeches, display more lexical repetitiveness than non-interpreted texts.

The lexical patterns of interpreted texts are related not only to cognitive load but also to the interpreting direction. Dayter (2018) examined the lexical features of a parallel SI corpus, SIREN, which consists of source texts in Russian and English, along with their respective simultaneous interpretations into English and Russian. The original and interpreted texts in the Russian and English subcorpora were compared. Although the interpreted Russian texts in the Russian subcorpus exhibited simplified features with lower lexical density and diversity, the English subcorpus displayed an opposite feature with the interpreted English texts being lexically denser and more diverse. Dayter (2018) proposed interpreting directionality as a possible explanation for the contradictory results between the two subcorpora: approximately 30% of English interpretations were performed by interpreters using their B language to interpret. Their frequent self-corrections contributed to a high proportion of content words and greater lexical variation.

Laviosa (1998) compared English translated texts and original English texts to investigate their lexical characteristics. The findings revealed that the translated texts exhibited simplification, with lower lexical density, a higher proportion of high-frequency words, and a higher repetition rate of the most commonly used words. To examine whether this simplified lexical pattern also applies to interpreted speeches, Li and Wang (2012) analyzed the lexical features of a self-built comparable corpus consisting of original English speeches and Chinese-into-English interpreted speeches on the topic of social life in Hong Kong. Their results corroborated the expected lexical features observed by Laviosa (1998), including reduced lexical density, a higher ratio of high-frequency words to low-frequency words, and increased recurrence of the most commonly used words, confirming the commonality of simplification in interpreted texts.

The reviewed literature indicates that the lexical features of interpreted speech are associated with both cognitive load and interpreting directionality. As SI is performed under stress and strain, it is reasonable to assume that experienced interpreters strategically and intentionally modify their lexical choices, potentially simplifying vocabulary (Lv and Liang, 2019), when their cognitive capacity is close to saturation. This adaptation helps to counteract the rising cognitive demands, mitigate cognitive burden and ensure smooth delivery and cognitive balance, causing the interpreted texts to exhibit simplification features characterized by a narrow vocabulary range and low information load (Laviosa, 1998). These findings further confirm Baker (1993) theory of translation universals.

### 2.7. Research gap, research aim, and research questions

Theoretical studies have confirmed the essential attributes of high cognitive demands of interpretation through model construction, while empirical studies have focused on measuring cognitive load, identifying triggering factors, and exploring their impact on interpreting performance. In terms of interpreting directionality, most studies have focused on the impact of interpreting direction on accuracy, fluency, and output quality, with the research subjects mainly consisting of novice or student interpreters and the interpreting mode studied primarily being consecutive interpreting. However, research has paid little attention to senior conference interpreters such as those employed by the UN, leading to insufficient studies on whether the cognitive load and directionality effects arising from SI can also influence the interpreting performance of UN simultaneous interpreters and how they respond to the heavy cognitive load during interpretation.

This study explored whether the cognitive load and directionality effects stemming from SI affect the lexical usage of UN simultaneous interpreters and what lexical strategies they adopt to reduce their cognitive load by analyzing the lexical features of non-interpreted English speeches and texts interpreted into English from Chinese and Russian in a self-built comparable corpus. This study addressed the following questions:

1. Are there differences between simultaneously interpreted texts and non-interpreted texts in terms of lexical features, as indicated by lexical density, lexical diversity, and lexical sophistication? If so, what are these differences?
2. Are there differences between A-into-B simultaneously interpreted texts and B-into-A simultaneously interpreted texts in terms of lexical features, as indicated by lexical density, lexical diversity, and lexical sophistication? If so, what are these differences?

## 3. Materials and methods

### 3.1. Corpora

This present study analyzed a specially-built comparable corpus with 409,084 tokens, consisting of speeches delivered in the United Nations Security Council. This comparable corpus is comprised of three subcorpora including non-interpreted speech texts in the US English, SubCorpusE, as well as interpreted texts from Chinese into English, SubCorpusC-E, which was the A-into-B interpreted texts, and interpreted texts from Russian into English, SubCorpusR-E, which was the B-into-A interpreted texts. Each subcorpus contains 160 texts, and the three subcorpora together comprise a total of 480 texts (Table 1).

**Table 1:** SubCorpora information.

SubCorpora	Task	Directionality	L1 of speakers/interpreters	Number of texts	Tokens
SubCorpusE	Non-interpreting	/	English	160	121912
SubCorpusC-E	Interpreting	A into B	Chinese	160	109642
SubCorpusR-E	Interpreting	B into A	English	160	177530

The comparable corpus is homogeneous in that (1) all the speeches in the corpus were delivered between January 2022 and May 2023, covering the same time span. The topics of these speeches are related to international security. As a result, the texts in the three subcorpora share a similar register and genre, characterized by a formal tone and diplomatic language conventions, and similar textual content due to their shared thematic emphasis on international security. (2) The interpreted speeches were performed by experienced interpreters working for the United Nations Security Council, with high bilingual proficiency and excellent interpreting skills, which ensures consistent interpreting quality across different interpreted texts. Therefore, it is reasonable to believe that the three subcorpora are comparable in linguistic style, textual content and interpreting quality.

### 3.2. Data collection

Lexical features were investigated in terms of lexical density, lexical diversity, and lexical sophistication. The data of these three indicators were obtained through a Corpus Tool, Sketch Engine, and an online software, vocabprofiles. In order to obtain lexical density values, all the texts in the three subcorpora were firstly uploaded to sketch engine. The content words of each text, such as nouns, verbs, adjectives, adverbs, numerals, and pronouns were identified by using sketch engine’s built-in POS (Part-of-Speech) tagging function and the number of tokens of each text was computed as well. As sketch engine does not have the function of distinguishing between lexical verbs and modal and auxiliary verbs, manual checking was performed to ensure the accurate identification of the lexical verbs, along with all the other content words for each text. After content words identification, the number of nouns, lexical verbs, adjectives, adverbs, numerals and pronouns, as well as the total number of content words for each individual text were computed separately. Lexical density scores of nouns, lexical verbs, adjectives, adverbs, numerals and pronouns for each text as well as the lexical density value of the whole text were correspondingly computed by excel according to the formula for calculating lexical density. For example, the noun density of each text is calculated by dividing the number of nouns in that text by the number of tokens in that text. Similarly, the lexical density of a text is the ratio of the total number of content words to the total tokens in that text.

Lexical diversity was analyzed by calculating the normalized TTR. First, the original TTR for each individual text was computed by vocabprofiles and the normalized TTR value per 1,000 tokens for each text was then computed by excel on the basis of the original TTR value and the length of each text. Lexical sophistication was investigated by measuring the proportions of uncommon and academic words appearing in each text. Two indices were used in this study: proportion of words in Academic Word List (AWL) (Coxhead, 2000) and proportion of off-list words, which are the words beyond the commonly used K1 and K2 word families (Coxhead, 1998). Similarly to TTR value, these two indices are influenced by the text length as well, so normalized values per 1,000 tokens for each text in terms of these two indices were computed to ensure a reliable comparison across different texts with different length.

### 3.3. Data analysis

In order to explore whether lexical features can be influenced by task complexity and interpreting directionality, the three subcorpora were re-grouped into two pairs. One pair (SubCorpusR-E versus SubCorpusE) is intended for comparing the 10 indices regarding lexical density, lexical diversity, and lexical sophistication between interpreted texts and non-interpreted texts. The reason for pairing these two subcorpora for comparison is that the speeches in both subcorpora were interpreted or delivered by native English speakers. This approach allows for better control of potential confounding variables such as interpreters or speakers with different first languages, thereby ensuring the validity of the study. The other pair (SubCorpuR-E versus SubCorpuC-E) is meant for comparing those 10 indices of lexical features between B-into-A and A-into-B interpreted texts. Descriptive statistics of measures for the two pairs are listed in Tables 2, 3. In addition, Jamovi was applied to perform two independent samples *T*-tests and the results are shown in Tables 4, 5.

**Table 2:** Descriptive statistics of measures for SubCorpusR-E and SubCorpusE.

Indices	Mean	SD	Task	SubCorpus
LD of text	35.49	4.90	Interpreting	SubCorpusR-E
	40.09	4.40	Non-interpreting	SubCorpusE
LD of nouns	17.66	2.69	Interpreting	SubCorpusR-E
	18.65	3.38	Non-interpreting	SubCorpusE
LD of lexical verbs	7.75	1.22	Interpreting	SubCorpusR-E
	9.42	1.04	Non-interpreting	SubCorpusE
LD of adjectives	6.74	1.55	Interpreting	SubCorpusR-E
	6.97	1.06	Non-interpreting	SubCorpusE
LD of adverbs	2.87	0.74	Interpreting	SubCorpusR-E
	2.96	0.76	Non-interpreting	SubCorpusE
LD of numerals	0.46	0.31	Interpreting	SubCorpusR-E
	0.61	0.37	Non-interpreting	SubCorpusE
LD of pronouns	1.17	0.37	Interpreting	SubCorpusR-E
	1.49	0.35	Non-interpreting	SubCorpusE
Normalized TTR	52.08	29.52	Interpreting	SubCorpusR-E
	69.49	29.61	Non-interpreting	SubCorpusE
Normalized proportion of AWL words	11.03	6.15	Interpreting	SubCorpusR-E
	13.65	6.92	Non-interpreting	SubCorpusE
Normalized proportion of off-list words	13.99	6.76	Interpreting	SubCorpusR-E
	20.58	7.65	Non-interpreting	SubCorpusE

LD, lexical density; TTR, type-token ratio; AWL, Academic Word List; off-list words, words beyond the commonly used K1 and K2 word families.

**Table 3:** Descriptive statistics of measures for SubCorpusR-E and SubCorpusC-E.

Indices	Mean	SD	Directionality	SubCorpus
LD of text	35.49	4.90	B-A	SubCorpusR-E
	42.22	12.35	A-B	SubCorpusC-E
LD of nouns	17.66	2.69	B-A	SubCorpusR-E
	20.68	12.02	A-B	SubCorpusC-E
LD of lexical verbs	7.75	1.22	B-A	SubCorpusR-E
	9.38	1.11	A-B	SubCorpusC-E
LD of adjectives	6.74	1.55	B-A	SubCorpusR-E
	7.59	1.13	A-B	SubCorpusC-E
LD of adverbs	2.87	0.74	B-A	SubCorpusR-E
	2.85	0.76	A-B	SubCorpusC-E
LD of numerals	0.46	0.31	B-A	SubCorpusR-E
	0.47	0.30	A-B	SubCorpusC-E
LD of pronouns	1.17	0.37	B-A	SubCorpusR-E
	1.25	0.43	A-B	SubCorpusC-E
Normalized TTR	52.08	29.52	B-A	SubCorpusR-E
	82.96	48.16	A-B	SubCorpusC-E
Normalized proportion of AWL words	11.03	6.15	B-A	SubCorpusR-E
	18.89	8.22	A-B	SubCorpusC-E
Normalized proportion of off-list words	13.99	6.76	B-A	SubCorpusR-E
	22.78	10.99	A-B	SubCorpusC-E

LD, lexical density; TTR, type-token ratio; AWL, Academic Word List; off-list words, words beyond the commonly used K1 and K2 word families.

**Table 4:** Summary of *t*-test on the 10 indices for SubCorpusR-E and SubCorpusE.

Indices	Statistic	df	*p*	Mean difference	SE difference	Effect size
LD of text	−8.851	318	<0.001	−4.6084	0.5207	−0.990
LD of nouns	−2.886	318	0.004	−0.9847	0.3412	−0.323
LD of lexical verbs	−13.252	318	<0.001	−1.6778	0.1266	−1.482
LD of adjectives	−1.543	318	0.124	−0.2294	0.1487	−0.173
LD of adverbs	−0.999	318	0.319	−0.0836	0.0837	−0.112
LD of numerals	−3.847	318	<0.001	−0.1467	0.0381	−0.430
LD of pronouns	−7.885	318	<0.001	−0.3176	0.0403	−0.882
Normalized TTR	−5.404	318	<0.001	−17.8622	3.3055	−0.604
Normalized proportion of AWL words	−3.578	318	<0.001	−2.6181	0.7318	−0.400
Normalized proportion of off-list words	−8.171	318	<0.001	−6.5944	0.8071	−0.913

LD, lexical density; TTR, type-token ratio; AWL, Academic Word List; off-list words, words beyond the commonly used K1 and K2 word families.

Test is significant at the 0.05 level (two-tailed).

**Table 5:** Summary of *t*-test on the 10 indices for SubCorpusR-E and SubCorpusC-E.

Indices	Statistic	df	*p*	Mean difference	SE difference	Effect size
LD of text	−6.407	318	<0.001	−6.73106	1.0506	−0.7163
LD of nouns	−3.097	318	0.002	−3.01494	0.9734	−0.3463
LD of lexical verbs	−12.533	318	<0.001	−1.63538	0.1305	−1.4013
LD of adjectives	−5.560	318	<0.001	−0.84513	0.1520	−0.6217
LD of adverbs	0.309	318	0.757	0.02588	0.0837	0.0346
LD of numerals	−0.215	318	0.830	−0.00725	0.0337	−0.0240
LD of pronouns	−1.936	318	0.054	−0.08681	0.0448	−0.2164
Normalized TTR	−6.916	318	<0.001	−30.88331	4.4657	−0.7732
Normalized proportion of AWL words	−9.691	318	<0.001	−7.86431	0.8115	−1.0835
Normalized proportion of off-list words	−8.619	318	<0.001	−8.79225	1.0201	−0.9636

LD, lexical density; TTR, type-token ratio; AWL, Academic Word List; off-list words, words beyond the commonly used K1 and K2 word families.

Test is significant at the 0.05 level (two-tailed).

## 4. Results

### 4.1. Descriptive and inferential data analysis for research question 1

Table 2 shows that SubCorpusR-E (interpreted texts) scored lower than SubCorpusE (non-interpreted texts) across all categories. Regarding lexical density, SubCorpusR-E presented a lower average textual density than SubCorpusE (*M* = 35.49, SD = 4.90 versus *M* = 40.09, SD = 4.40), as well as lower average densities for nouns, lexical verbs, adjectives, adverbs, numerals, and pronouns. The same pattern emerged in the indices of lexical diversity and sophistication. The three indices: normalized TTR (*M* = 52.08, SD = 29.52 versus *M* = 69.49, SD = 29.61), normalized proportion of AWL words (*M* = 11.03, SD = 6.15 versus *M* = 13.65, SD = 6.92), and normalized proportion of off-list words (*M* = 13.99, SD = 6.76 versus *M* = 20.58, SD = 7.65) suggested that the interpreted texts exhibited lower average values than the non-interpreted texts. These results indicate that SubCorpusR-E had lower lexical density, less lexical diversity, and less lexical sophistication than SubCorpusE.

Table 4 shows that the interpreted texts exhibited significantly different lexical features from the non-interpreted texts in the following indices: LD of text (*t*(318) = −8.851, *p* < 0.001, MD = −4.6084), LD of nouns (*t*(318) = −2.886, *p* < 0.05, MD = −0.9847), LD of lexical verbs (*t*(318) = −13.252, *p* < 0.001, MD = −1.6778), LD of numerals (*t*(318) = −3.847, *p* < 0.001, MD = −0.1467), LD of pronouns (*t*(318) = −7.885, *p* < 0.001, MD = −0.3176), normalized TTR (*t*(318) = −5.404, *p* < 0.001, MD = −17.8622), normalized proportion of AWL words (*t*(318) = −3.578, *p* < 0.001, MD = −2.6181), and normalized proportion of off-list words (*t*(318) = −8.171, *p* < 0.001, MD = −6.5944). However, only two indices did not show a significant difference, which were LD of adjectives (*t*(318) = −1.543, *p* = 0.124, MD = −0.2294) and LD of adverbs (*t*(318) = −0.999, *p* = 0.319, MD = −0.0836). This finding suggests that there was no significant difference in the lexical density of modifying parts of speech such as adjectives and adverbs between interpreted texts and non-interpreted texts.

### 4.2. Descriptive and inferential data analysis for research question 2

Table 3 compares the 10 linguistic indices between two sets of interpreted texts: B-into-A interpreted texts versus A-into-B interpreted texts. SubCorpusR-E (B-into-A) generally scored lower than SubCorpusC-E (A-into-B). In terms of lexical density, SubCorpusR-E exhibited lower average densities than SubCorpusC-E for overall texts (*M* = 35.49, SD = 4.90 versus *M* = 42.22, SD = 12.35), as well as for nouns, lexical verbs, adjectives, adverbs, numerals, and pronouns. Similarly, lexical diversity and sophistication were also lower in the B-into-A interpreted texts compared with the A-into-B interpreted texts, which was reflected by the mean values of normalized TTR (*M* = 52.08, SD = 29.52 versus *M* = 82.96, SD = 48.16), normalized proportion of AWL words (*M* = 11.03, SD = 6.15 versus *M* = 18.89, SD = 8.22), and normalized proportion of off-list words (*M* = 13.99, SD = 6.76 versus *M* = 22.78, SD = 10.99). These results suggest that the texts in SubCorpusR-E were less textually dense, less lexically diverse, and less lexically sophisticated compared with those texts in SubCorpusC-E.

Table 5 indicates that there were significant differences between the B-into-A interpreted texts and the A-into-B interpreted texts in the following indices: LD of text (*t*(318) = −6.407, *p* < 0.001, MD = −6.73106), LD of nouns (*t*(318) = −3.097, *p* < 0.05, MD = −3.01494), LD of lexical verbs (*t*(318) = −12.533, *p* < 0.001, MD = −1.63538), LD of adjectives (*t*(318) = −5.560, *p* < 0.001, MD = −0.84513), normalized TTR (*t*(318) = −6.916, *p* < 0.001, MD = −30.88331), normalized proportion of AWL words (*t*(318) = −9.691, *p* < 0.001, MD = −7.86431), and normalized proportion of off-list words (*t*(318) = −8.619, *p* < 0.001, MD = −8.79225). However, there were no significant differences between the two sets of interpreted texts in the following three indices: LD of adverbs (*t*(318) = 0.309, *p* = 0.757, MD = 0.02588), LD of numerals (*t*(318) = −0.215, *p* = 0.830, MD = −0.00725), and LD of pronouns (*t*(318) = −1.936, *p* = 0.054, MD = −0.08681).

## 5. Discussion

### 5.1. Simplified vocabulary features in interpreted texts

The findings of this study highlight the presence of lower vocabulary density, diminished lexical diversity, and reduced lexical complexity in the interpreted texts compared with the non-interpreted texts. Eight of the 10 indices examined were significantly lower in the interpreted texts, namely noun density, lexical verb density, numeral density, pronoun density, lexical density of texts, normalized TTR, normalized proportion of AWL words, and normalized proportion of off-list words. These results indicate that interpreted texts exhibit simplified vocabulary features. One possible explanation is that professional interpreters deliberately use specific interpreting strategies to cope with the substantial cognitive load involved in interpretation.

During the listening and analysis stage, interpreters often commit structure-related information to working memory to reduce their input load (Lv and Liang, 2019). SI, working in real time and in parallel, requires interpreters to closely adhere to the linguistic characteristics of the source text (Ma and Cheung, 2020). This adherence to linear constraints results in a higher proportion of function words and, consequently, a lower lexical density in the interpreted speech. In addition, the substantial influx of source language information poses significant cognitive challenges for interpreters; therefore, they often purposefully simplify their vocabulary, opting for commonly used and stereotypical words that have a high frequency of occurrence. This deliberate choice leads to a decrease in lexical variety and sophistication in the interpreted product, helping to mitigate processing difficulties (Lv and Liang, 2019). This finding in terms of lexical simplification responds to the notion that simultaneous interpreters, burdened by heavier cognitive load and constrained by time limitations, may inhibit the generation of complicated and lengthy sentence structures, resulting in a simplification of syntax (Liu et al., 2023).

### 5.2. Simplified vocabulary features in B-into-A interpreted texts

The findings of this study indicate that B-into-A interpreted texts demonstrate lower lexical density, limited vocabulary range, and decreased lexical complexity compared with A-into-B interpreted texts. Seven of the 10 indices studied were significantly lower in the B-into-A interpreted texts, namely noun density, lexical verb density, adjective density, lexical density of texts, normalized TTR, normalized proportion of AWL words, and normalized proportion of off-list words. These results indicate that B-into-A interpreted texts present simplified lexical features.

Previous research has acknowledged the presence of an interpreting directionality effect, but there is no consensus on which interpreting directionality imposes a higher cognitive load. The prevailing belief is that interpreting from one’s A language into one’s B language entails higher cognitive demands and is more challenging due to the need to find equivalent expressions in one’s B language. However, this study offers a different perspective, with the B-into-A interpreted texts displaying significantly simplified lexical features. As lexical simplification in SI can alleviate the burden on working memory, freeing up more capacity for other cognitive tasks (Plevoets and Defrancq, 2018), as discussed earlier, it is reasonable to believe that simultaneous interpreters working in the B-into-A direction consciously use this strategy to manage the cognitive demands of SI. This also suggests that the B-into-A interpreting direction does not have lower cognitive demands, at least not lower than the A-into-B direction, thereby calling into question the long-standing belief that interpreting into one’s B language is more difficult.

An alternative perspective worth considering is that SubCorpusC-E (consisting of A-into-B interpreted texts) demonstrated relatively higher lexical density, diversity, and complexity than SubCorpusR-E. These characteristics can be attributed to the inherent properties of the Chinese and English languages. Chinese has a tendency to favor high-context and implicit communication, while English leans toward low-context and explicit expression (Hall, 1976). Consequently, when interpreting from Chinese into English, interpreters are required to make implicit source language information explicit by paraphrasing, summarizing, explaining, and providing additional details, resulting in the presence of denser, more varied, and more complex lexical features in A-into-B interpreted texts.

### 5.3. Effective cognitive load management in experienced interpreters

This research emphasizes that experienced simultaneous interpreters working for the UN, notwithstanding their exceptional bilingual skills and excellent interpreting expertise, still face a cognitive load stemming from the inherent cognitive demands of the interpreting task and different interpreting directions. However, compared with novice interpreters, they have the conscious ability to use interpreting strategies, prioritize multiple cognitive demands and manage their cognitive load while operating with limited cognitive resources (Liu et al., 2004). They adeptly allocate their attentional resources during the coordination process (Darò, 1989), achieving an equilibrium between cognitive demand and cognitive capacity. The presence of both simple and rich lexical features is compelling evidence of their proficient cognitive load management.

### 5.4. Limitations and future studies

This study has certain limitations that should be acknowledged. First, the analysis focused on 10 lexical feature indices, which may not cover all possible indicators and did not investigate the diversity and sophistication of specific word classes. As a result, the results offered a general lexical feature of interpreted texts without a detailed examination of the diversity and complexity within specific word categories. Future research should aim to incorporate a broader range of vocabulary feature metrics and undertake a more comprehensive and systematic investigation of the lexical characteristics of interpreted texts. Furthermore, this study provides evidence that interpreters are able to use effective lexical strategies to combat cognitive overload during SI. However, to what extent these strategies are conscious actions taken by interpreters or automatic cognitive operations acquired through professional training is a matter that warrants further exploration in cognitive psychology. Additionally, apart from lexical strategies, it is worthwhile to investigate the various strategies used by interpreters to cope with the cognitive challenges associated with different interpreting modes and directions. Finally, this study adheres to the conventional practices of comparable corpus studies, with a particular emphasis on the comparison of the target language texts without considering the impact of the parameters such as linguistic characteristics, discourse structure, stylistic elements, delivery rate and text difficulty specific to the source languages. This limitation is inherent not only in the current research but also in the broader scope of comparable corpus studies. Future studies will incorporate parallel corpora and consider multiple factors such as source languages, interpreting mode and context, and interpreters’ expertise to conduct a more comprehensive analysis of lexical features.

## Data availability statement

The raw data supporting the conclusions of this article will be made available by the authors, without undue reservation.

## Author contributions

LZ: Conceptualization, Data curation, Investigation, Methodology, Writing – original draft, Writing – review & editing. DJ: Project administration, Writing – review & editing.

## Abbreviations

SI: simultaneous interpreting
A language: first language, native language, dominant language
B language: second language, non-native language, non-dominant language
L1: first language
L2: second language
LD: lexical density
TTR: type-token ratio
AWL: Academic Word List
Off-List Words: words beyond the commonly used K1 and K2 word families
